# Online Fault Detection of Permanent Magnet Demagnetization for IPMSMs by Nonsingular Fast Terminal-Sliding-Mode Observer

**DOI:** 10.3390/s141223119

**Published:** 2014-12-05

**Authors:** Kai-Hui Zhao, Te-Fang Chen, Chang-Fan Zhang, Jing He, Gang Huang

**Affiliations:** 1 School of Traffic and Transportation Engineering, Central South University, Changsha 410073, China; E-Mails: zhaokaihuicn@gmail.com (K.-H.Z.); ctfcyt@163.com (T.-F.C.); gangder@csu.edu.cn (G.H.); 2 School of Electrical and Information Engineering, Hunan University of Technology, Zhuzhou 412007, China; E-Mail: hejing@263.net; 3 School of Information Science and Engineering, Central South University, Changsha 410073, China

**Keywords:** nonsingular fast terminal-sliding-mode observer (NFTSMO), interior permanent magnet synchronous motors (IPMSMs), permanent magnet (PM), demagnetization, fault detection, flux-weakening control

## Abstract

To prevent irreversible demagnetization of a permanent magnet (PM) for interior permanent magnet synchronous motors (IPMSMs) by flux-weakening control, a robust PM flux-linkage nonsingular fast terminal-sliding-mode observer (NFTSMO) is proposed to detect demagnetization faults. First, the IPMSM mathematical model of demagnetization is presented. Second, the construction of the NFTSMO to estimate PM demagnetization faults in IPMSM is described, and a proof of observer stability is given. The fault decision criteria and fault-processing method are also presented. Finally, the proposed scheme was simulated using MATLAB/Simulink and implemented on the RT-LAB platform. A number of robustness tests have been carried out. The scheme shows good performance in spite of speed fluctuations, torque ripples and the uncertainties of stator resistance.

## 1. Introduction

Permanent magnet synchronous motors (PMSMs) are widely used as motors in electric vehicles, electrical traction systems, industrial applications, wind generation and defense, due to their high energy efficiency, high torque-to-weight ratio, high power factor, fast response, rugged construction and reliable operation [1–3]. However, permanent magnet (PM) demagnetization faults often occur in the practical application of PMSMs by flux-weakening control and, in severe cases, even cause irreversible demagnetization [4]. Therefore, it is important to improve the reliability for the PMSM by online monitoring of PM flux-linkage and analysis of PM demagnetization.

To prevent PM demagnetization, many solutions [5–7] have been proposed for optimizing the magnetic circuit and, thus, reducing the risk of PM demagnetization from the motor-design standpoint; this may lead to increasing manufacturing cost, but can reduce maintenance costs. This approach is referred to as the static prevention method. On the other hand, a dynamic monitoring method can provide accurate PM flux-linkage information by online monitoring of the PMSM control system, which can effectively prevent the occurrence of more serious demagnetization and decrease the extent of irreversible demagnetization.

Xiao *et al.* [8] have proposed a dynamic method to estimate PM flux-linkage for a surface-mounted permanent magnet synchronous motor (SPMSM) based on an extended Kalman filter (EKF), which provides a reference for online monitoring of the PM flux-linkage in the PMSM closed-loop control system. Shi *et al.* [9] have achieved PM flux-linkage identification for an IPMSM using an EKF.

Sliding-mode variable structure control [10–12] has good robustness against system parameter perturbations, external disturbances and inaccurate mathematical models, but the chattering phenomenon of traditional sliding-mode control limits its application. Terminal-sliding-mode [13] (TSM) is a new sliding-mode control method that can design a nonlinear sliding manifold to converge in finite time, but it has singularity problems. Feng *et al.* [14,15] proposed a nonsingular terminal-sliding-mode (NTSM) control approach, which solves the singularity problem of the traditional TSM and offers high tracking accuracy in the steady state. However, it converges slowly far away from the equilibrium point and has chattering problems when designing a control law combined with global reach conditions, which also limit its practical application. Levant [16] proposed a high-order sliding-mode (HOSM) control, which applies discontinuous control on a higher time derivative of the sliding-mode manifold to eliminate chattering while retaining the good properties of the traditional sliding-mode.

### 1.1. Contribution

The main contribution of this study is to propose a robust nonsingular fast terminal-sliding-mode observer (NFTSMO) to detect PM flux-linkage demagnetization faults for interior permanent magnet synchronous motors (IPMSMs) by flux-weakening control. PM demagnetization faults are reconstructed by the NFTSMO, which is combined with the chattering elimination characteristics of high-order sliding-mode and the fast convergence of nonsingular fast terminal-sliding-mode. It is robust to speed fluctuations, torque ripples and stator resistance uncertainties. The fault-decision criteria and the fault-processing method are also presented.

### 1.2. Structure of This Article

In Section 2, a mathematical model of rotor PM flux-linkage demagnetization for IPMSMs in the *d*-*q* synchronous reference frame is introduced. In Section 3, an NFTSMO is designed to reconstruct the PM flux-linkage for an IPMSM, and the stability of the observer is proven. The fault-decision criteria and fault-processing method for IPMSM by flux-weakening control are also presented. The simulation and experimental results are shown in Section 4. Finally, conclusions are given.

## 2. IPMSM Mathematical Model of PM Flux-Linkage in Normal and Demagnetization

### 2.1. IPMSM Mathematical Model

The stator voltage Equations for a PMSM in the *d*-*q*-axis reference frame are as follow:

(1) $$\{\begin{array}{cc} u_{d} & =R_{s}i_{d}+\frac{d\psi_{d}}{dt}-\omega_{e}\psi_{q}\\ u_{d} & =R_{s}i_{q}+\frac{d\psi_{q}}{dt}+\omega_{e}\psi_{d} \end{array}$$

where *u_d_*, *u_q_* are the *d-q*-axis voltages, *i_d_*, *i_q_* are the *d-q*-axis stator currents, *ψ_d_*, *ψ_q_* are the stator flux-linkage, *R_s_* is stator resistance, *ω_e_* is electrical angular velocity, respectively.

The stator flux-linkage equations for an IPMSM in the *d-q*-axis reference frame are:

(2) $$\{\begin{array}{ll} \psi_{d} & =L_{d}i_{d}+\psi_{r}\\ \psi_{q} & =L_{q}i_{q} \end{array}$$

where *ψ_r_* is the rotor PM flux-linkage and *L_d_*, *L_q_* are the *d-q*-axis inductances.

Substituting Equation (2) into Equation (1), the equations for IPMSM in the *d-q*-axis reference frame can be expressed as follows:

(3) $$\{\begin{array}{ll} \frac{di_{d}}{dt} & =\frac{u_{d}}{L_{d}}-\frac{R_{s}}{L_{d}}i_{d}+\omega_{e}\frac{L_{q}}{L_{d}}i_{q}\\ \frac{di_{q}}{dt} & =\frac{u_{q}}{L_{q}}-\frac{R_{s}}{L_{q}}i_{q}-\omega_{e}\frac{L_{d}}{L_{q}}i_{d}-\omega_{e}\frac{\psi_{r}}{L_{q}} \end{array}$$

### 2.2. IPMSM Mathematical Model of PM Demagnetization

**Figure 1.**
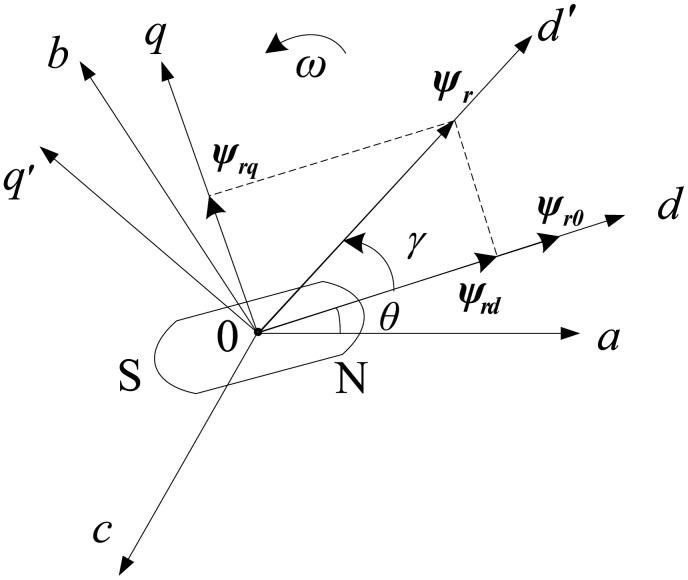
Variation of interior permanent magnet synchronous motor (IPMSMs) PM flux-linkage.

In the PMSM operation, the PM flux magnitude and direction can vary in a very wide range due to the position feedback signal error or the impact of external effects, such as temperature rise and poor working conditions. This influences the system control performance and may even lead to PM demagnetization. In this case, there is a deviation angle *γ* between the directions of rotor flux and the *d*-axis of the *d-q* reference frame. The PM flux-linkage *ψ_r_* will produce the new component *ψ_rd_* and *ψ_rq_* in the *d-q*-axes [8], respectively. It is illustrated in Figure 1.

Then, the stator flux-linkage Equations (2) for IPMSM are formed as follows:

(4) $$\{\begin{array}{ll} \psi_{d} & =L_{d}i_{d}+\psi_{rd}\\ \psi_{q} & =L_{q}i_{q}+\psi_{rq} \end{array}$$

where *ψ*_*rq*_ =*ψ*_*r*_ sin *γ*,*ψ*_*rd*_ =*ψ*_*r*_ cos *γ*.

Substituting Equation (4) into Equation (1) and considering that the time constant of the mechanical system is much larger than that of the electrical system in the PMSM, that is *dψ_r_*/*dt* ≈ 0, *dψ_rd_*/*dt* ≈ 0, *dψ_rq_*/*dt* ≈ 0. The equations for IPMSM in the *d*-*q*-axis reference frame can be rearranged as follows:

(5) $$\{\begin{array}{ll} \frac{di_{d}}{dt} & =-\frac{R_{s}}{L_{d}}i_{d}+\omega_{e}\frac{L_{q}}{L_{d}}i_{q}+\frac{u_{d}}{L_{d}}+\omega_{e}\frac{\psi_{rq}}{L_{d}}\\ \frac{di_{q}}{dt} & =-\frac{R_{s}}{L_{q}}i_{q}-\omega_{e}\frac{L_{d}}{L_{q}}i_{d}+\frac{u_{q}}{L_{q}}-\omega_{e}\frac{\psi_{rd}}{L_{q}} \end{array}$$

## 3. PM Demagnetization Fault Detection by NFTSMO

In order to achieve good performances, such as fast convergence and better tracking precision, to solve the problem of the NTSM converges slowly far away from the equilibrium point, a nonsingular fast terminal-sliding-mode observer (NFTSMO) is proposed to detect a PM demagnetization fault.

### 3.1. Design and Analysis Stability of the NFTSMO

According to Equation (5), the mathematical model of the IPMSM can be designed as follows:

(6) $$\dot{\text{x}}=\text{Ax}+\text{Bu}+\text{Dd}$$

where: ***x*** = [*i_d_ i_q_*]*^T^* are state vectors, ***u*** = [*u_d_ u_q_*]*^T^* are input vectors, ***d*** = [*ψ_rd_ ψ_rq_*]*^T^* are the reconfigurable vectors of the PM demagnetization fault, and:

$$A=\left[\begin{array}{cc} -\frac{R_{s}}{L_{d}} & \omega_{e}\frac{L_{q}}{L_{d}}\\ -\omega_{e}\frac{L_{d}}{L_{q}} & -\frac{R_{s}}{L_{q}} \end{array}\right],B=\left[\begin{array}{cc} \frac{1}{L_{d}} & 0\\ 0 & \frac{1}{L_{q}} \end{array}\right],D=\left[\begin{array}{cc} 0 & \frac{\omega_{e}}{L_{d}}\\ -\frac{\omega_{e}}{L_{q}} & 0 \end{array}\right]$$

According to Equation (6), the observer can be designed as follows:

(7) $$\dot{\overset{ˆ}{\text{x}}}=\text{A}\hat{x}+\text{Bu}+\upsilon$$

where: ***x̂*** = [*i̇̂_d_ i̇̂_q_*]*^T^*, “ˆ” denotes the estimated values, ***υ*** = [*υ_d_ υ_q_*]*^T^* is the control input vector of the observer.

Then, the stator current error equation can be obtained by subtracting Equation (6) from Equation (7):

(8) ė=Ae+Dd−υ

where: ***e*** = ***x*** − ***x̂*** [*x*_1_ − *x̂*_2_ *x*_2_ − *x̂*_2_]*^T^* = [*e*_1_ *e*_2_]*^T^* are stator current errors in the *d*-*q*-axis reference frame.

According to the high-order sliding-mode control and the definition of system relative degree [16], the relative degree of system (8) is one; the system can eliminate the chattering by second-order or more than a two order sliding-mode control.

The traditional second-order nonsingular terminal-sliding-mode (NTSM) manifold is designed as follows [14]:

(9) $$\text{l}=\text{s}+\beta {ṡ}^{p/q}$$

where: ***l*** = [*l*_1_ *l*_2_]*^T^*, ***s*** = [*s*_1_ *s*_2_]*^T^* = ***e*** = [*e*_1_ *e*_2_]*^T^*, ***β***= *diag*(*β*_1_, *β*_2_), *β*_1_ > 0, *β*_2_ > 0, 1 < *p*/*q* < 2, *p* > 0, *q* > 0, *p* and *q* are odd.

This paper proposes the following second-order nonsingular fast terminal-sliding-mode (NFTSM) manifold inspired by [17]:

(10) $$\text{l}=a\text{s}+\text{b}ṡ+\beta {ṡ}^{p/q}$$

where: ***l*** ∈ ***R***^2^, ***l*** = [*l*_1_ *l*_2_]*^T^*, ***s*** = [*s*_1_ *s*_2_]*^T^* = ***e*** = [*e*_1_ *e*_2_]*^T^*, ***β*** = *diag*(*β*_1_, *β*_2_), *β*_1_ > 0, *β*_2_ > 0, *a* > 0, *b* > 0, 1 < *p*/*q* < 2, *p* > 0, *q* > 0, *p* and *q* are all odd.

#### Remark 1

*According to NFTSM manifold (10), it determines the convergence phase by judging the size of* ‖***s***‖. *The values of a, b are as follows*:

(11) $$\{\begin{array}{ll} a=m_{1},b=n_{1} & \left\|\text{s}\right\|\geq \sigma \\ a=m_{2},b=n_{2} & \left\|\text{s}\right\|<\sigma \end{array}$$

when ‖***s***‖ > *σ*, the linear sliding-mode plays a main role, and it can accelerate moving to the sliding-mode manifold; when ‖***s***‖ < *σ*, the nonsingular sliding-mode plays a main role: it converges to zero in finite time. Therefore, it has the advantages both of the linear sliding-mode and nonsingular terminal-sliding-mode.

#### Remark 2

*It can regulate the convergence speed of **l** by choosing a, b in the linear sliding-mode phase and regulate the convergence speed of **l** by choosing **β**, **p** and **q** in the nonsingular terminal-sliding-mode phase*.

Then, the robust HOSM control law is designed to ensure that the system states always move towards the NFTSM manifold and the system is robust to parameter uncertainties and external disturbances. The HOSM control law of the observer is designed according to the following theorem.

##### Theorem 1

*The stator current error Equation (8) can converge to zero in finite time, if the NFTSM manifold is chosen as Equation (10) and the control law (12) is designed as follows*:

(12) $$\upsilon =\upsilon_{eq}+\upsilon_{n}$$

*where*:

(13) $$\upsilon_{eq}=\text{Ae}$$

(14) $$\upsilon_{n}=\int_{0}^{t}\left[\frac{aṡ}{(p/q)\beta {ṡ}^{p/q-1}+b}+(k+\eta )\mathrm{sgn}(\text{l})+\mu \text{l}\right]d\tau$$

where *k* > max (***D*** ‖***ḋ***‖), *k* > 0, *η* > 0, *μ* > 0 are the designed parameters.

###### Proof

The following Lyapunov function is selected to be:

(15) $$V(t)=\frac{1}{2}{\text{l}}^{T}\text{l}$$

Differentiating *V* with respect to time, one obtains:

(16) $$\begin{array}{ll} \dot{V}(t) & ={\text{l}}^{T}\text{i}={\text{l}}^{T}(a\dot{\text{s}}+\text{b}\ddot{s}+(\text{p}/\text{q})\beta {\dot{\text{s}}}^{p/q-1}\ddot{s})\\ & ={\text{l}}^{T}\left[(p/q)\beta {\dot{\text{s}}}^{p/q-1}+b\right]\left[\ddot{\text{s}}+\frac{a\dot{\text{s}}}{(p/q)\beta {\dot{\text{s}}}^{p/q-1}+b}\right] \end{array}$$

From the stator current error Equation (8) and the Equations (12)–(13), we get:

(17) $$ė=\text{Ae}+\text{Dd}-\upsilon =\text{Dd}-\upsilon_{n}$$

From Equation (17), Equation (16) can be rearranged as follows:

(18) $$\dot{V}(t)={\text{l}}^{T}[(\text{p}/\text{q})\beta {ṡ}^{p/q-1}+\text{b})][\text{D}\overset{˙}{d}-(k+\eta )\mathrm{sgn}(\text{l})-\mu \text{l}]$$

Since the parameter *k* satisfies *k* > max (***D***‖***ḋ***‖), then *V̇*(*t*) can be expressed as:

(19) $$\begin{array}{ll} \dot{V}(t) & \leq -{\text{l}}^{T}\left[(p/q)\beta {ṡ}^{p/q-1}+b\right][\eta \mathrm{sgn}(\text{l})+\mu \text{l}]\\ & =-\left[(p/q)\begin{array}{c} \min\\ i=1,2 \end{array}(\beta_{i}{ṡ}_{i}^{p/q-1})+b\right]\;\left[\eta \|\text{l}\|+\mu {\left\|\text{l}\right\|}^{2}\right] \end{array}$$

Taking *p* and *q* as all odd and 1 < *p*/*q* < 2, e.g., *q* = 2*m* + 1, *p* = 2*m* + 3, *m* ∈ *N*, this gives:

(20) $${ṡ}_{i}^{p/q-1}={ṡ}_{i}^{(p-q)/q}={\left({ṡ}_{i}^{2}\right)}^{\left(p-q)/(2q\right)}={\left({ṡ}_{i}^{2}\right)}^{1/(2m+1)}\geq 0$$

We get:

(21) $$(p/q)\begin{array}{c} \min\\ i=1,2 \end{array}(\beta_{i}{ṡ}_{i}^{p/q-1})+b>0$$

Substituting Equation (21) into Equation (19), one of the following two conditions exists:

- ***V̇*** < 0, for ‖***l***‖ ≠ 0. The condition for Lyapunov stability is satisfied. The system states can reach the sliding mode ***l*** = 0 within finite time.
- ***V̇*** = 0, for ‖***l***‖ = 0. This shows that the system states have reached the sliding-mode manifold ***l*** = 0.

Therefore, the system states can reach the NFTSM manifold ***l*** within finite time. After ***l*** reaches zero in finite time, both ***s*** and ***ṡ*** will also reach zero in finite time; the system will stay on the second-order sliding mode ***s*** = ***ṡ*** = 0 [14,15]. Then, the stator current error Equation (8) will converge to zero in finite time. This completes the proof.

#### Remark 3

*Considering the assumption of the demagnetization model (5), that is:* ***ḋ*** = [*ψ̇_rd_ ψ̇_rq_*] ≈ 0, so ***D*** ‖***ḋ***‖ ≈ 0. *We can find that positive constants ksatisfy k* > 0, *such that the state estimation errors converge to zero infinite time*.

#### Remark 4

*Because of using the second-order sliding-mode technique, it can be seen from Equations (12)-(14) that the control* ***υ*** *is continuous and smooth, which can be used to estimate the PM demagnetization fault directly*.

### 3.2. Reconfiguration of PM Demagnetization Fault

When the stator current error Equation (8) stays on the second-order sliding-mode manifold, it satisfies ***e***= ***ė*** = 0, according to the sliding-mode equivalent control method [18]. Substituting this into Equation (8), the following equivalent PM flux-linkage can be obtained:

(22) Dd=υ

That is,

(23) $$\{\begin{array}{ll} {\hat{\psi }}_{rd} & =-\frac{1}{\omega_{e}}L_{q}\upsilon_{q}\\ {\hat{\psi }}_{rq} & =\frac{1}{\omega_{e}}L_{d}\upsilon_{d} \end{array}$$

Therefore, the amplitude of rotor PM flux-linkage *ψ̂_r_* can then be estimated:

(24) $${\hat{\psi }}_{r}=\sqrt{{\hat{\psi }}_{rd}^{2}+{\hat{\psi }}_{rq}^{2}}$$

**Figure 2.**
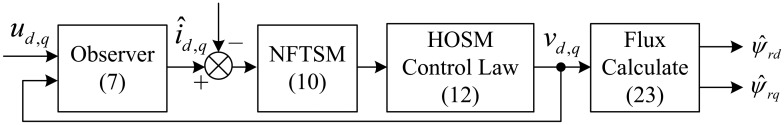
Principle diagram of the nonsingular fast terminal-sliding-mode observer (NFTSMO) for the reconfiguration of the PM flux-linkage.

The principle diagram of the proposed NFTSMO for the reconfiguration of the PM demagnetization fault is described in Figure 2.

### 3.3. PM Demagnetization Fault Detection and Fault Processing

To estimate the severity of the PM demagnetization fault, it is necessary to define a severity factor (diagnosis index) λ. The severity factor λ is defined by:

(25) $$\lambda =\frac{\psi_{r}-{\hat{\psi }}_{r}}{\psi_{r}}$$

where *ψ̂_r_* is the estimated value of PM flux-linkage *ψ_r_* in a control period using Equation (24). The low diagnosis index λ means that the estimation error is small, and the PMSM is in the normal condition. If the severity factor λ is greater than the threshold value Δλ, then the PMSM is under a demagnetization fault.

**Figure 3.**
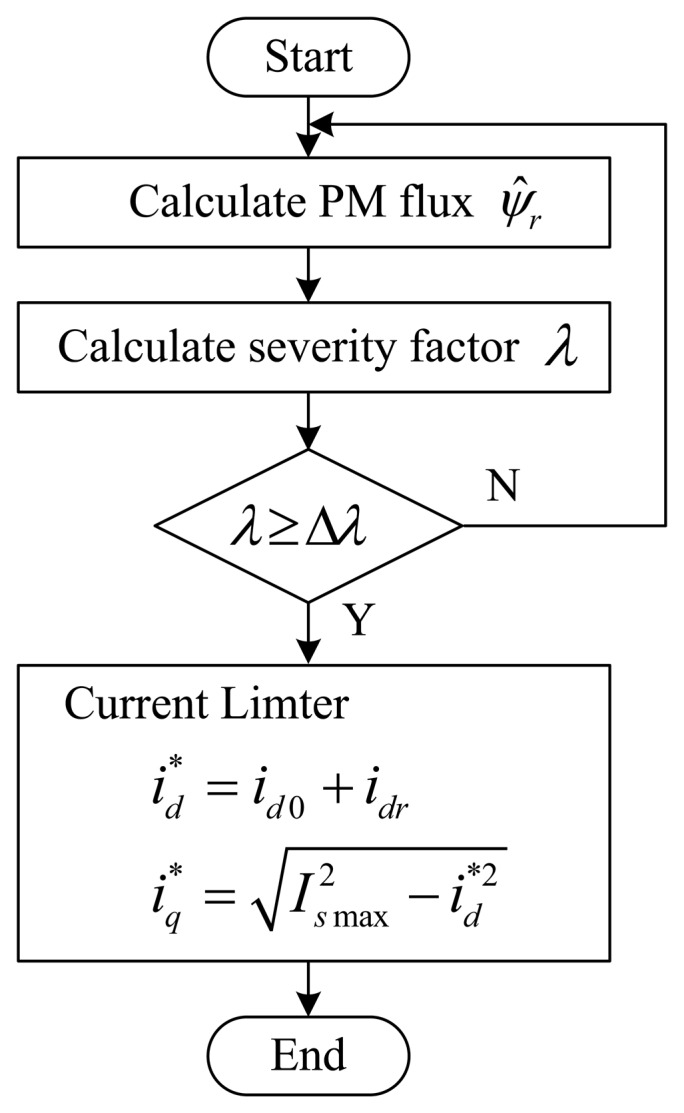
Flowchart of demagnetization fault diagnosis and fault treatment.

Figure 3 shows the flowchart of the proposed fault diagnosis and fault treatment. For the first step, the PM flux-linkage *ψ̂_r_* is calculated using Equation (24). In the second step, the severity factor λ is calculated using Equation (25). The third step is the demagnetization fault decision using the diagnosis index λ. The final step is fault processing. If severity factor λ is greater than the upper bound of threshold value Δλ, then the current limiter is activated.

**Figure 4.**
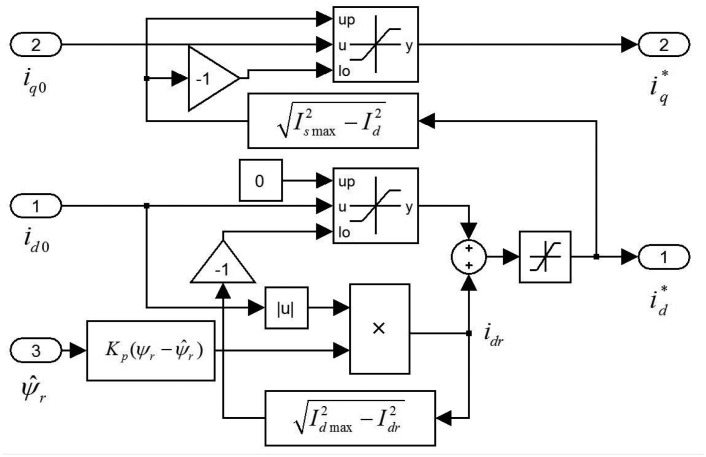
Control block diagram of the current limiter.

In order to avoid the drop of the operating point below the kneepoint and at the same time using the flux-weakening control, it is necessary to properly limit the $i_{d}^{*}$. The current limiter is designed to prevent the dropping of the operating point below the knee point and achieving the minimum torque ripple. Figure 4 is the schematic diagram of the proposed current limiter [19]. In Figure 4, *I_smax_* is the maximum phase current, and *I_dmax_* is the maximum allowed current of the *d*-axis. The compensation current *i_dr_* is a positive direct current, and it is obtained by inputting the weakening current *i_d_*_0_, with a compensation function given by:

(26) $${\text{i}}_{dr}=\rho \lambda |{\text{i}}_{d0}|=K_{p}(\psi_{r}-{\hat{\psi }}_{r})|i_{d0}|$$

where *ρ* is the compensation coefficient, *K_p_* = *ρ*/*ψ_r_*. The *i_dr_* is the input of a current limiter that is bound to the references $i_{d}^{*}$ and $i_{q}^{*}$. The appropriate coefficient *K_p_* can reduce the *d*-axis current *i_d_* and increase the *q*-axis current *i_q_*; thus, it can reduce the PM demagnetization risk and reduce the torque ripple significantly.

## 4. Simulations and Experiments

**Figure 5.**
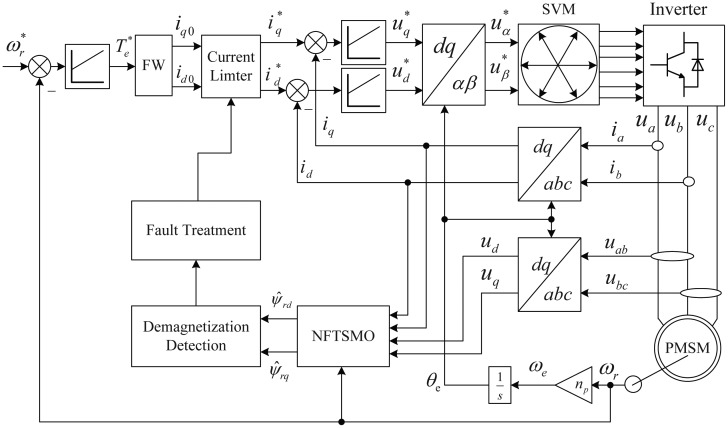
Schematic diagram of online PM demagnetization fault-detection system for IPMSM.

The proposed PM demagnetization fault-detection scheme has been simulated using MATLAB/Simulink and implemented on the RT-LABplatform. A schematic diagram of the online PM demagnetization fault-detection system for IPMSM is shown in Figure 5. The flux-weakening control strategy is carried out on the IPMSM [20]. The IPMSM parameters are listed in Table 1. This section evaluates the dynamic performance of the proposed NFTSMO, which is compared with the sliding-mode observer (SMO) and the nonsingular terminal-sliding-mode observer (NTSMO).

### 4.1. Simulations

The proposed NFTSMO is designed according to Theorem 1. The observer parameters are chosen as follows: *p* = 7, *q* = 5, *β*_1_ = *β*_2_ = 0.1, *k* + *η* = 3000, *μ* = 2000. The initial values of *i̇̂_d_*, *i̇̂_q_* are set to 1.5 A, 1.5 A. The parameters *a*, *b* and *σ* of NFTSM manifold are chosen as:

(27) $$\{\begin{array}{lll} a=60, & b=1 & \left\|\text{s}\right\|\geq 0.1\\ a=1, & b=0.0001 & \left\|\text{s}\right\|<0.1 \end{array}$$

To observe the dynamic performance of the proposed scheme, the initial rotor speed is set to 500 rpm and subsequently increases to 1000 rpm at 1 s; the initial value of the load torque is set to 0 Nm and subsequently increases to 2 Nm at 2 s; and the initial value of the stator resistance is set to 2.875 Ω and subsequently increases to 5.75 Ω at 3 s.

Because the actual dynamic process of PM demagnetization in an IPMSM is very difficult to simulate, the approach of setting initial values of the PM flux-linkage amplitude and the rotor deviation angle [8] are utilized. These can be used to verify the validity of the observer.

The initial value of the PM flux-linkage amplitude is set to 0.175 Wb and subsequently decreases to 0.10 Wb at 4 s. The initial value of the deviation angle γ is set to 0° and subsequently increases to 30° at 5 s. The upper bound Δλ of the severity factor λ is set to 0.25.

**Figure 6.**
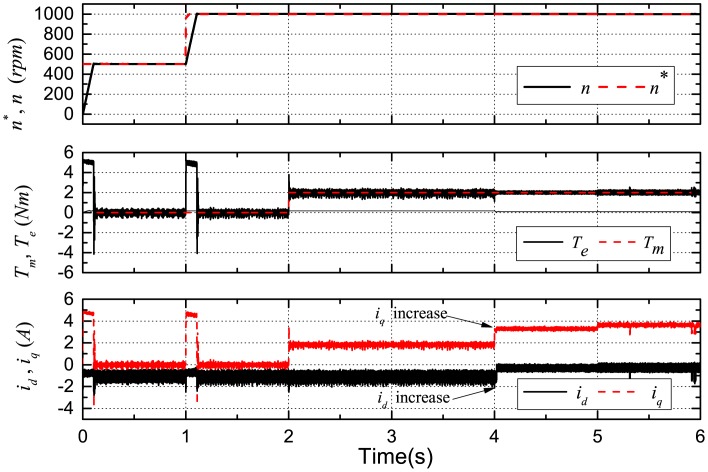
Simulation results: the reference, actual speed and torque; the actual *d*-*q*-axis currents.

The reference, actual rotor speed, the reference, actual torque and the actual *d*-*q*-axis stator currents are shown in Figure 6. n* is the reference speed, *n* is the actual speed, *T_m_* is the reference load torque and *T_e_* is the actual IPMSM output torque.

**Figure 7.**
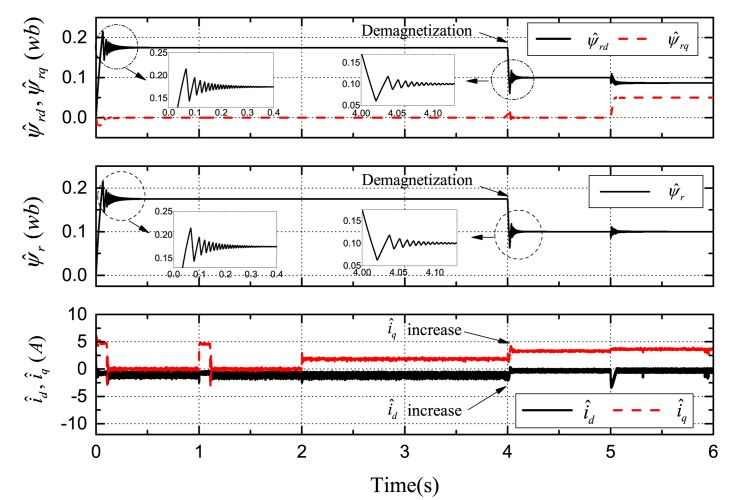
Simulation results: demagnetization fault detection by sliding-mode observer (SMO).

**Figure 8.**
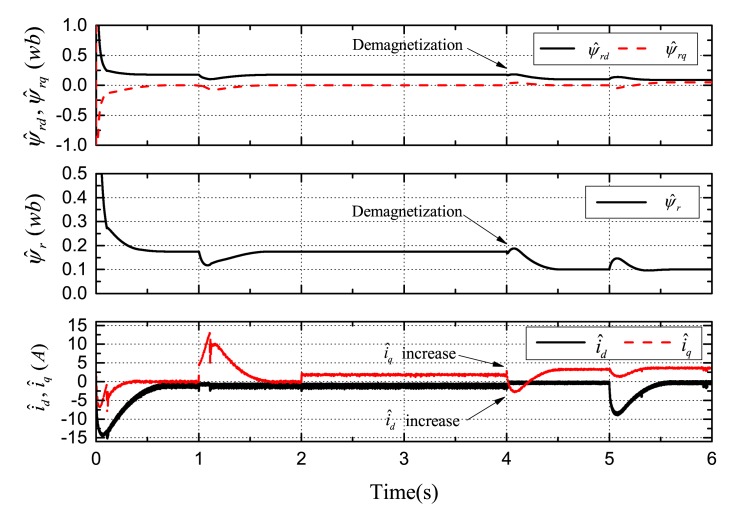
Simulation results: demagnetization fault detection by nonsingular terminal-sliding-mode observer (NTSMO).

**Figure 9.**
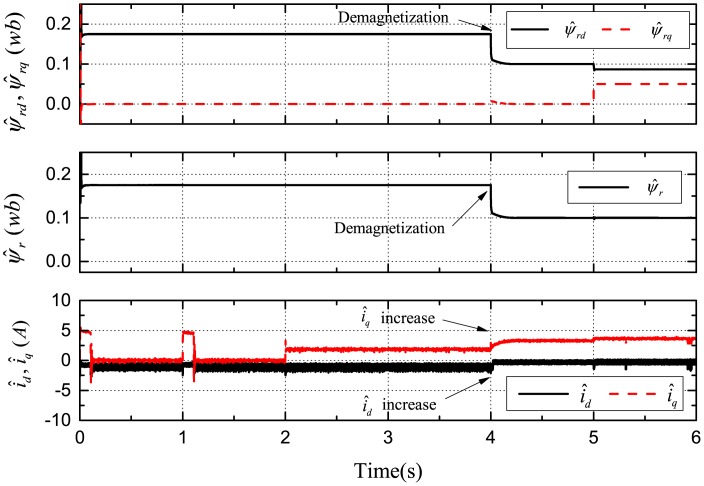
Simulation results: demagnetization fault detection by NFTSMO.

Figures 7, 8 and 9 demonstrate the simulation results of demagnetization fault detection by the SMO, NTSMO, and NFTSMO. They indicate the estimated *d*-*q*-axis PM flux-linkage, PM flux amplitude and *d*-*q*-axis stator currents.

The following conclusions can be drawn from these simulation results:

- Figure 7 demonstrates that the SMO has the chattering phenomenon when moving to the sliding-mode manifold. Figure 8 demonstrates that the NTSMO converges slowly far away from the equilibrium point. It can be seen from Figure 9 that the NFTSMO converges quickly, which has the advantages of both SMO and NTSMO.
- When the PM flux-linkage amplitude is decreased to 0.10 Wb at 4 s, the severity factor is λ = (*ψ_r_* ‒ *ψ̂_r_*)/*ψ_r_* = (0.175 − 0.10)/0.175 = 0.4286. This is greater than the upper bound Δλ, and therefore, the current limiter is activated. The absolute value of *d*-axis current *i_d_* (*i_d_* < 0 by flux-weakening control) is decreased, that is the *d*-axis current *i_d_* increases, while the *q*-axis current *i_q_* increases; and then, this can reduce PM demagnetization risk and torque ripple significantly.
- When the deviation angle γ is increases to 30 ° at 5 s, the estimated *d*-axis flux *ψ̂_rd_* is 0.0865 Wb; the estimated *q*-axis flux *ψ̂_rq_* is 0.0500 Wb; and the estimated flux amplitude *ψ̂_r_* is 0.0999 Wb; whereas, the actual *d*-axis flux is *ψ_rd_* = 0.10 × *cos*30° = 0.0866 Wb, and the actual *q*-axis flux is *ψ_rq_* = 0.10 × *sin*30° = 0.0500 Wb. It can be seen from simulation results that the estimated PM flux-linkages are very close to the actual values.

### 4.2. Experimental Results

RT-LAB is a powerful, modular, distributed, real-time platform from OPAL-RT Technologies, Inc. It supports model-based design using rapid control prototyping (RCP) and hardware-in-the-loop simulation (HILS) of complex dynamic systems. HILS differs from pure real-time simulation and RCP by the use of the real controller in the loop; this controller is connected to the rest of the system simulated by RT-LAB. Therefore, in HILS, the plant is simulated, but the controller is real [21].

**Figure 10.**
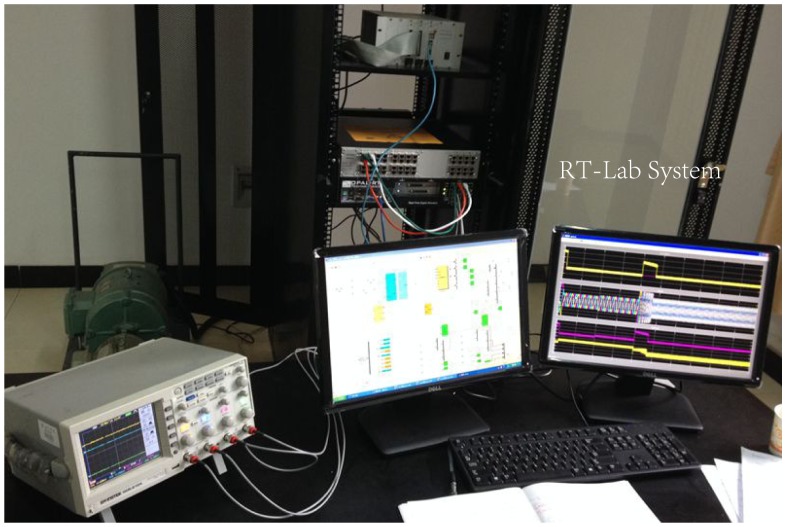
RT-Labplatform.

**Figure 11.**
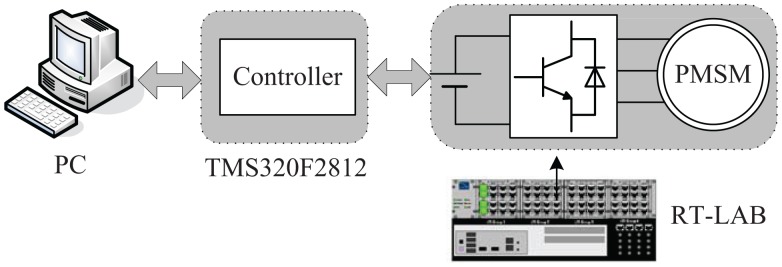
Configuration of the RT-Lab hardware-in-the-loop simulation (HILS) system.

To implement the proposed approach, experiments have been carried out on an OP5600 RT-Lab platform using HILS. The RT-Labplatform is shown in Figure 10, and the configuration is shown in Figure 11.

The controller is a TMS320F2812 digital signal processor, which implements high-performance control and computation. The IGBT inverter model uses blocks from the RT-Events toolbox to achieve precise modeling of the switching signals, which are done through the interpolation scheme embedded in RT-Events. The PWM (Pulse Width Modulation) switching frequency is chosen as 5 kHz. The sampling period is chosen as 50 *μ*s, which are the same as the SMO, NTSMO, NFTSMO and IPMSM.

**Figure 12.**
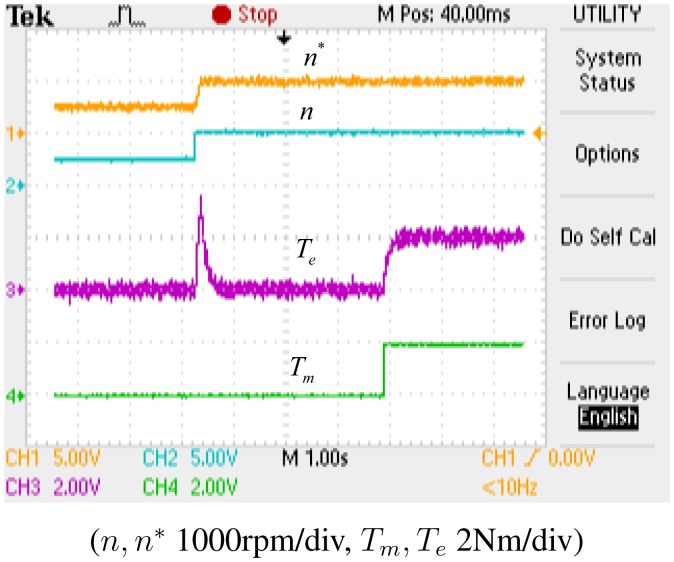
Experimental results: the reference, actual rotor speed and torque.

Figure 12 shows the reference rotor speed (n*), the actual rotor speed (*n*), the reference torque (*T_m_*) and the actual torque (*T_e_*). As shown in Figure 12, the actual speed (*n*) can track the reference speed (n*) precisely in the steady state, whereas the actual torque (*T_e_*) fluctuates when the speed increases from 500 rpm to 1000 rpm, but it converges to the reference torque (*T_m_*) quickly.

**Figure 13.**
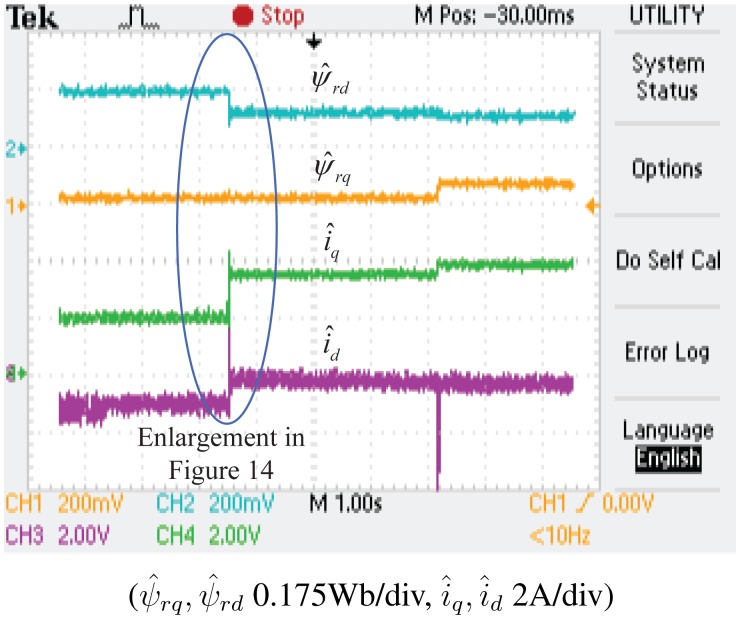
Experimental results: demagnetization fault detection by SMO.

**Figure 14.**
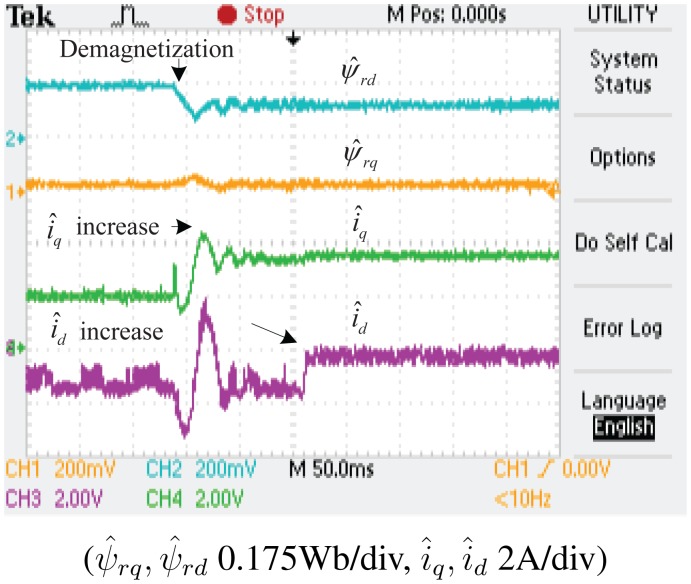
Enlargement of Figure 13.

**Figure 15.**
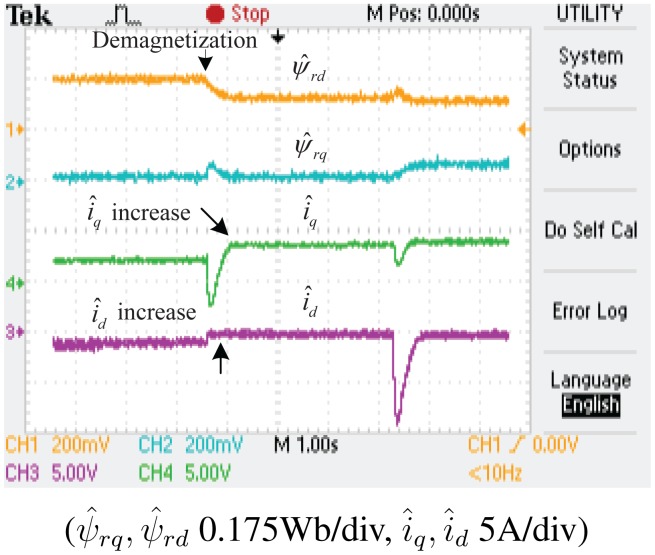
Experimental results: demagnetization fault detection by NTSMO.

**Figure 16.**
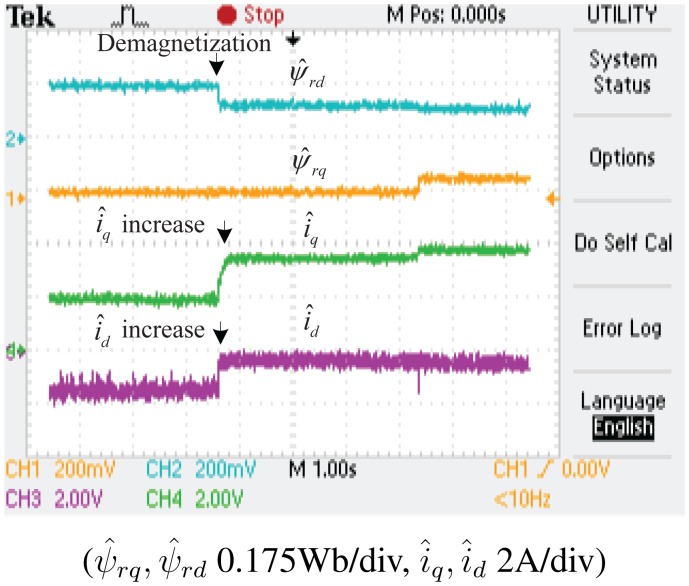
Experimental results: demagnetization fault detection by NFTSMO.

The experimental results of the demagnetization fault-detection using SMO, NTSMO and NFTSMO are shown in Figures 13, 14, 15 and 16. These figures indicate the estimated values of the *d-q*-axis PM flux-linkages and *d-q*-axis stator currents.

The following conclusions can be drawn from these experimental results:

- When the PM flux-linkage amplitude is decreased to 0.10 Wb, the severity factor λ becomes greater than the upper bound Δλ, and therefore, the current limiter is activated. The *d*-axis current *i_d_* is increased, which can reduce PM demagnetization risk. The *q*-axis current *i_q_* is also increased, which can reduce torque ripple significantly.
- When the deviation angle γ of PM flux-linkage is increased to 30 °, the estimated value of *d*-axis flux *ψ̂_rd_* is decreased to 0.0864 Wb, whereas that of *q*-axis flux *ψ̂_rq_* is increased to 0.0500 Wb, and the estimated PM flux-linkage amplitude *ψ̂_r_* is 0.0998 Wb. The estimated PM flux-linkages are very close to the actual value.
- Figure 14 is a larger version of Figure 13, which demonstrates that the SMO has the chattering phenomenon. Figure 15 demonstrates that the NTSMO converges slowly far away from the equilibrium point. Figure 16 demonstrates that the proposed NTSMO has good tracking performance and strong robustness against parameter fluctuations.

Simulation and experimental results have demonstrated that the proposed nonsingular fast terminal-sliding-mode observer converges quickly and has low sensitivity to parameter variations.

## 5. Conclusions

An IPMSM mathematical model of demagnetization has been introduced. A robust nonsingular fast terminal-sliding-mode observer (NFTSMO) has been constructed to simulate permanent magnet (PM) demagnetization faults for IPMSMs. The fault decision criteria and the fault-processing method have been presented. The overall scheme has been simulated using MATLAB/Simulink and implemented on the RT-LAB platform. A number of robustness tests have been carried out. The results verify the efficiency and stability of the proposed scheme. This method can accurately estimate PM flux-linkage.

The scheme shows good performance in spite of speed fluctuations, torque ripples and the uncertainties of stator resistance. The online fault detection scheme of PM demagnetization for IPMSMs by flux-weakening control can prevent the deterioration of demagnetization and reduce the extent of irreversible demagnetization.

## Figures and Tables

**Table 1.** Parameters of IPMSM.

**Parameters**	**Unit**	**Values**
Rated power (*P_N_*)	*KW*	2
Rated voltage (*U_N_*)	*V*	380
Rated current (*I_N_*)	*A*	4
Rated speed (*n_N_*)	*r/min*	1000
Stator resistance (*R_s_*)	Ω	2.875
*q*-axis inductance (*L_q_*)	*H*	0.0075
*d*-axis inductance (*L_d_*)	*H*	0.0025
Rotational Inertia (*J*)	*kg · m*^2^	0.0008
Rotor PM flux (*ψ_r_*)	*Wb*	0.175
Number of pole pairs (*n_p_*)	pairs	4
